# Evaluation of unique identifiers used as keys to match identical publications in Pure and SciVal – a case study from health science

**DOI:** 10.12688/f1000research.8913.2

**Published:** 2016-09-06

**Authors:** Heidi Holst Madsen, Dicte Madsen, Marianne Gauffriau

**Affiliations:** 1 Faculty Library of Natural and Health Sciences, Copenhagen University Library, The Royal Library, Copenhagen, DK-2200, Denmark; 2 Copenhagen Business School Library, Frederiksberg, DK-2000, Denmark; 3 SUND Research & Innovation, Faculty of Health and Medical Sciences, University of Copenhagen, Copenhagen, DK-2200, Denmark

**Keywords:** match key, unique identifier, DOI, Scopus ID, PubMed ID, Pure, SciVal, bibliometric analysis

## Abstract

Unique identifiers (UID) are seen as an effective key to match identical publications across databases or identify duplicates in a database. The objective of the present study is to investigate how well UIDs work as match keys in the integration between Pure and SciVal, based on a case with publications from the health sciences. We evaluate the matching process based on information about coverage, precision, and characteristics of publications matched versus not matched with UIDs as the match keys. We analyze this information to detect errors, if any, in the matching process. As an example we also briefly discuss how publication sets formed by using UIDs as the match keys may affect the bibliometric indicators number of publications, number of citations, and the average number of citations per publication.

The objective is addressed in a literature review and a case study. The literature review shows that only a few studies evaluate how well UIDs work as a match key. From the literature we identify four error types: Duplicate digital object identifiers (DOI), incorrect DOIs in reference lists and databases, DOIs not registered by the database where a bibliometric analysis is performed, and erroneous optical or special character recognition.

The case study explores the use of UIDs in the integration between the databases Pure and SciVal. Specifically journal publications in English are matched between the two databases. We find all error types except erroneous optical or special character recognition in our publication sets. In particular the duplicate DOIs constitute a problem for the calculation of bibliometric indicators as both keeping the duplicates to improve the reliability of citation counts and deleting them to improve the reliability of publication counts will distort the calculation of average number of citations per publication.

The use of UIDs as a match key in citation linking is implemented in many settings, and the availability of UIDs may become critical for the inclusion of a publication or a database in a bibliometric analysis.

## Introduction

Unique identifiers (UIDs) have been introduced for more and more entities, e.g. Open Researcher and Contributor ID (ORCID) for researchers, and digital object identifiers (DOI) for research publications, etc. One advantage of UIDs is that integrations between databases potentially can be done much more efficiently. This is stressed in a recent evaluation of metrics in research evaluations ( Wilsdon *et al.*, 2015) p. 15–22, 145).

The purpose of the present study is to find out how well UIDs work as match keys for publications and thus to create publication sets for bibliometric analysis. Traditionally, this is done via a match key based on bibliographic information such as author, title, etc. The exact method is rarely described. An exception is the evaluation of the Danish Council for Independent Research ( Schneider *et al.*, 2014, p. 36–38).

**Figure 1.**
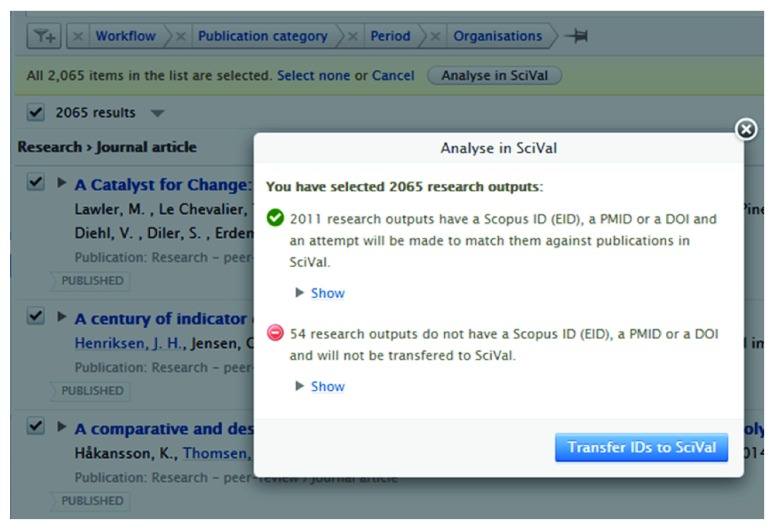
Screenshot from Pure. Publication set automatically analyzed for UIDs before export to SciVal, August 2015. Source: Pure from Elsevier, version 4.23.1, local installation at University of Copenhagen.

UIDs are simple match keys compared to the traditional method (e.g. Olensky *et al.*, 2015). We explore how the method works in the integration between the current research information system (CRIS), Pure, and the bibliometric research evaluation tool, SciVal, ( Elsevier, 2014). SciVal builds on data from the citation index Scopus, and Pure provides a uniform identification of researchers and the organizational structure at a university. UIDs make it easy to export a publication set from Pure to SciVal for bibliometric analysis ( Figure 1). An alternative is to define the publication set, e.g. the publications from a department, in Scopus or Web of Science (WoS). This is often a resource-demanding task as researchers do not always register their affiliations correctly and consistently in publications (e.g. Moed *et al.*, 1995, p. 390).

A widely used UID for research publications is the DOI. It was launched in 2000 ( International DOI Foundation, 2015, sec. 1.2) and is by now assigned to publications by more than 5,000 publishers from the big players, e.g. Elsevier, to small societies, e.g. Danish Chemical Society ( CrossRef, 2015b). Other UIDs for research publications such as arXiv ID from 1991 ( arXiv.org, 2015), PubMed ID (PMID) from 1997 ( Ostell *et al.*, 1998, p. 27), and Scopus ID (EID) from 2004 ( Elsevier, 2004) are not as prevalent as the DOI. The UIDs are not assigned to the publication itself (the content), but to the metadata of a publication. How and if this metadata is recorded can differ between databases.

The integration between Pure and SciVal was launched in October 2014 and DOI, PMID and EID were used as match keys. From October 2015 Elsevier matches publications in Pure with Scopus and attributes EIDs to publications in Pure. And from March 2016 the integration between Pure and SciVal is based on EID alone. The latter will not affect the present study as we analyze publication sets downloaded in August and December 2015. It means that our study is a documentation of the functionality of the first matching processes between Pure and SciVal and our results can serve as background for new evaluations. Is the new matching process based on EIDs better? We do not know. EIDs from Scopus are added to publications in Pure and these publications can subsequently be matched with SciVal which builds on Scopus. It would be interesting to see if this matching process solves some of the problems of having UIDs as match keys which we show in this study or if it causes new problems.

## Objective

The objective of the present study is to investigate how well UIDs work as match keys in the integration between Pure and SciVal, based on a case with publications from the health sciences. We evaluate the matching process based on information about coverage, precision, and characteristics of publications matched versus not matched with UIDs as the match keys. We analyze this information to detect errors, if any, in the matching process.

The matching of publications across databases can be used to create publication sets for bibliometric analysis. As an example we also briefly discuss how publication sets formed by using UIDs as the match keys may affect the bibliometric indicators number of publications, number of citations, and the average number of citations per publication.

### Limitations

We analyzed a case study from the health sciences and our results should be interpreted in context of the case. The results cannot be generalized, e.g. to other research areas. Furthermore we analyzed information on coverage, precision, and characteristics to detect errors in the matching process between Pure and SciVal. This type of information did not allow us to detect all types of errors. If a publication was assigned a wrong UID in one of the databases and thus wrongly matched we may not have detected this. In a sample of the apparently correctly matched publications we checked if the matched publications were identical and found no errors.

Throughout the study we use the term publication even though we did not analyze the full content of a publication but only parts of its metadata representations in Pure and SciVal; mainly UIDs, publication type, language, and journal. If a publication had more UIDs assigned to it in one of the databases we assumed that each of the UIDs represented the same publication. We do not know the details of the matching process between Pure and SciVal, but assumed that if one UID did lead to a match then the other UIDs assigned to the same publication were not taken into account. From SciVal we did not get any reports on contradicting UIDs for a publication. Such contradictions could question if there was a match or not.

We describe the characteristics of two publication sets from Pure and the two matched publication sets from SciVal, but it is beyond the scope of this paper to do a thorough analysis of the metadata quality of the Pure and SciVal publication sets. Also we limit the discussion of bibliometric indicators to three basic bibliometric indicators. More advanced indicators and their construction in SciVal are not discussed.

## Methods

The objective was addressed in a literature review and a case study. The literature review gave us an indication of the use of UIDs as keys for matching identical publications in one or more databases, and an overview of the precision of the method. As the integration between Pure and SciVal is relatively new and evaluations are not yet reported in the literature, we conducted our own case study to see if the implementation of UIDs as match keys between SciVal and Pure confirms what other studies have found.

### Literature review

Evaluations of UIDs as match keys to detect identical publications in different databases or duplicates in one database were identified. Information on search terms, search strategy and databases are given below. UIDs as match keys have been used for many applications, but our focus was on research publications, with a particular interest in how the method may affect bibliometric analysis. Thus, the search was limited to studies where UIDs of publications are used as the match keys or part of the match key, and in which the method is analyzed and discussed in some detail.

An exploratory search showed that the terminology for applying match keys to detect identical publications is not consistent. It is called citation linking or reference linking or simply matching or linking. Matching within the same database is called deduplication. The term citation matching is also used, but often for the more specific purpose where citing and cited publications are matched. We also saw examples of more general terminology, namely integration or interoperability between databases or retrieval strategy. In our subsequent searches the different terms for applying match keys to identify identical publications were combined (Boolean AND) with different terms for UID: unique, identifier, DOI, PMID. This gave us an idea of which databases use UIDs as match keys, e.g. CrossRef, Mendeley, and Altmetric.com. We also included these databases as search terms and combined them with the different terms for UIDs.

The searches were conducted in WoS ( https://login.webofknowledge.com/), Scopus ( https://www.scopus.com/), and Google Scholar ( https://scholar.google.dk/). No range of years was specified. If no relevant publications were found in WoS and Scopus, we continued the search in Google Scholar. This means that not only peer-reviewed research but also a few reports were included in the literature review. In relevant publications, we manually scanned references and citations for other relevant publications. The searches were done in August and September 2015 followed by later supplementary searches based on the references found in August and September.

### Case study

In the case study we explored information about coverage, precision, and characteristics of publications matched versus not matched in the integration between Pure and SciVal to detect errors, if any, in the matching process. Our publication set is from the Department of Clinical Medicine (DoCM) at University of Copenhagen (UCPH). DoCM registers approximately 2,000 research publications in the UCPH Pure database per year. The majority are peer-reviewed journal publications in English. As this type of publication and the health sciences are well-covered in Scopus/SciVal ( Mongeon & Paul-Hus, 2016, p. 218–219+222; Valderrama-Zurián *et al.*, 2015, p. 570–571), we expected the DoCM publication set to be well-fitted for our purpose, namely to explore the matching process, rather than how well Scopus/SciVal covers publications from a department.

The publication set was limited to research publications published in 2014, registered and validated in Pure. Publications published before 2014 were not included as these have been validated at department or group level and the data quality is not consistent as no common practice was in place. The validation of publications from 2014 was undertaken by the authors of this article and three information specialists from the University Library as a service for the Faculty of Health and Medical Sciences. As part of the validation process, language and publication type was determined according to the categories available in Pure. This information is utilized in the Results section. However, the focus of the validation was not UIDs as match keys, and fields for UIDs were not mandatory. If PMID or EID was registered in Pure, it is most likely because the publication was imported from PubMed or Scopus. A publication in Pure without a UID may not have a UID, or the UID is simply not registered in Pure. As mentioned in the Introduction, from October 2015 Elsevier matches publications in Pure with Scopus and attributes EIDs to new publications and retrospectively.

Our choice of case implies some limitations. The publication sets have too few non-journal publications to draw conclusions on their coverage and the precision in publications matched versus not matched with UIDs as match keys. Furthermore, the publication year 2014 gives the publications too short of a time since publication to obtain robust citation counts.

The case study alone did not lead to generalizable results, but the results were compared to findings from the literature review to identify trends and compatibility with previous studies.

Before we analyzed the outcome of the matching process based on UIDs as match keys, we downloaded, merged, and cleaned data from Pure and SciVal. This process was carried out in August 2015 (n=2068) and repeated in December 2015 (n=2066). It is possible for researchers and administrative staff to make retrospective changes to the registrations in Pure; this is the most plausible explanation for the lower number of publications in December.

Data software

• Pure local installation at University of Copenhagen, version 4.22.1 for the August download and version 4.23.1 for the December download (data download)
• SciVal June 8, 2015, and September 30, 2015 releases (data analysis and download)
• Microsoft Excel 2007 (data cleaning and analysis)

Data download, merging and cleaning

Raw data was downloaded from Pure in August and December 2015 using the following filters:

• Organisational unit = Department of Clinical Medicine
• Publication category = Research
• Publication statuses and dates > Latest > Date: Selected range = 2014
• Workflow = Validated

To fit relevant data in just one worksheet in Excel and be able to create a .csv file, most of the data columns were deleted, and only the following kept:

• Access to electronic version (full text) > DOI (Digital Object Identifier)-0
• journalAssociation.title
• pages
• persons[0].lastName
• typeClassification.typeClassification
• title
• id [=Pure ID]
• Source[sourceId]: PubMed [=PMID]
• Source[sourceId]: Scopus [=EID]
• language.language

Due to an error in the Copenhagen University Pure at the time, it was not possible to download a full data report of publications with the DOI column. Instead, first an ungrouped raw data report was downloaded, then the same report grouped on DOI. The two reports were matched on Pure ID to create one list with DOI data where available.

The Data set 1 DoCM Pure data August.csv and Data set 2 DoCM Pure data December.csv files comprise our "raw" Pure data – ever so slightly tidied to a) create one full data report with DOI where available, b) fit relevant columns in one worksheet to be able to create a .csv file.

The Pure "raw" data was furthermore cleaned by:

• Removing superfluous spaces at the end of DOIs to be able to match DOIs in the Pure data with the DOIs in the SciVal data.

After the Pure data was sent to SciVal for analysis, the resulting SciVal publication sets (August and December) were downloaded from SciVal with the following information:

• Title
• Authors
• Journal title
• Citations
• Pages
• DOI
• Publication-type
• EID [=Scopus ID]
• PubMed ID [=PMID]

The Data set 3 DoCM SciVal data August.csv and Data set 4 DoCM SciVal data December.csv files comprise our raw SciVal data.

The SciVal raw data was furthermore cleaned by:

• Removing "2-s2.0-" from the EIDs to be able to match with the EIDs in the Pure data.
• Duplicate DOIs were identified to remove superfluous/irrelevant publications:
  • Article vs. Article in Press (Article kept in data set)
  • Publication duplicates (if one duplicate had a PMID, that is the one we kept; otherwise we randomly selected which duplicate to keep)
  • Publication vs. publication attributed wrong ID in Scopus/SciVal and not occurring in the Pure data set (Publication in Pure data set kept)
  • Publication registered as one publication type vs. same publication registered as another publication type (duplicate with same publication type as in the Pure data set was kept)
  • Author's reply (not in Pure data set) having same DOI as the publication (in Pure data set) it relates to (publication in Pure data set kept).

A note on some Article in Press occurrences in the SciVal data:

1. Sometimes SciVal imports only the Article in Press instance of an article in Scopus (instead of the published article instance), or the article is registered in Scopus only as Article in Press, although it is published.
2. During an automatic update in June 2015 of the UCPH Pure, a number of validated publications were changed from published in 2015 to published in 2014, although really they were published in 2015. As such, they should not have been part of our Pure publication set to begin with.

In the Results section, characteristics for three groups of publications are shown: Publications with UID exported from Pure to SciVal and matched, publications with UID exported from Pure to SciVal and not matched, and publications without UID not exported from Pure to SciVal. The publications without UID (DOI, PMID, or EID) were extracted from the cleaned Data set 1 and 2 with Pure data. To identify publications exported from Pure to SciVal and matched, we compared UIDs (DOI, PMID, or EID) in the cleaned Data set 3 with UIDs in the cleaned Data set 1 (August download). Publications in Data set 1 with no corresponding UID in Data set 3 constitute publications with UID exported from Pure to SciVal and not matched. This was repeated for Data set 2 and 4 (December download). The EIDs attributed automatically to publications in Pure were not visible in our raw data. We found 32 publications in Data set 4 from SciVal which must have an EID in Pure and SciVal as no other UID was assigned to them. Finally we compared UIDs in the cleaned Data set 3 and 4 to identify publications matched in SciVal in December but not in August.

## Results

### Results of the literature review

The literature review shows two trends. Firstly, the publication year of relevant studies is 2011 or later. Older UIDs such as arXiv ID and PMID do not seem to have the same momentum as DOI. Secondly, the use of UIDs as match keys in bibliometric studies and citation indexes seems under-reported. A possible explanation is that the commercial players do not publish their methodologies in full detail ( Olensky, 2014, p. 3). However, in a study from 2015, two bibliometric research groups provide documentation for how they use DOI as part of their match keys ( Olensky *et al.*, 2015, p. 7–9).

If we do not focus on the matching process in citation indexes and bibliometric analysis alone, we find an increasing number of tools for handling and analyzing research publications, e.g. CrossRef’s cited-by links ( CrossRef, 2015a) and Altmetric.com’s embeddable badges ( Altmetric.com, n.d.). Evaluations of these databases were also included in our literature review. But also for these tools evaluations of UIDs as match keys are rare.

We included 17 studies in the literature review. Two of the studies’ main focus was to evaluate the integration of UIDs in existing systems and are in that sense comparable to our objective. For a small publication set, Zahedi *et al.* (2014) evaluated the quality of Mendeley metadata by a comparison with Web of Science metadata. The DOIs did not match for 15 publications (8%). Missing DOI or erroneous special character recognition were identified as error types. Franceschini *et al.* (2015) showed that a DOI in Scopus can point to multiple not identical publications. These error types were included in the overview in Table 1.

**Table 1.** Precision - types of errors.

Error due to	Reported by
Duplicate DOIs	( Costas *et al.*, 2015, p. 2015) ( Zahedi *et al.*, 2014a, p. 1495) ( Haustein *et al.*, 2014, p. 1) ( Franceschini *et al.*, 2015)
Incorrect DOIs in reference lists and databases	( Franceschini *et al.*, 2013, p. 2153) ( Costas *et al.*, 2015, p. 2015) ( Zahedi *et al.*, 2014a, p. 1495)
DOIs not registered by the database where a bibliometric analysis is performed	( Haustein & Siebenlist, 2011, p. 449) (Bar-Ilan *et al.*, 2012, p. 101) ( Franceschini *et al.*, 2014, p. 759) ( Zahedi *et al.*, 2014b)
Erroneous optical or special character recognition	( Haustein & Siebenlist, 2011, p. 449) ( Zahedi *et al.*, 2014b)

The rest of the studies used UIDs as match keys for different purposes and in doing so discussed how well the matching process worked. Different from our study, the main focus regarding the UIDs was the coverage. For all the studies, UIDs did not cover all publications potentially relevant for the analyses. Some studies only included publications with UIDs, and others supplemented the match keys with other types of bibliographic information to include publications without UIDs. We provide a short summary of how UIDs were used in the studies. Keep in mind that UIDs are used in many other studies and tools, but with very limited or no documentation and discussion of the UIDs.

The vast majority of the studies (ten) analyzed altmetric scores or altmetric methods. They used UIDs (DOI, PMID, and arXiv ID) sometimes in combination with other match key elements to match a publication set from WoS, Scopus, arXiv.org, a local database, or specific journals with identical publications in Mendeley, Almetric.com, Twitter, Wikipedia, Impact Story, CiteULike, BibSonomy, Connotea, or a selection of Elsevier databases (Bar-Ilan *et al.*, 2012; Costas *et al.*, 2014; Haunschild & Bornmann, 2016; Haustein *et al.*, 2014; Haustein & Siebenlist, 2011; HEFCE, 2015; Kraker *et al.*, 2015; Nuredini & Peters, 2015; Zahedi *et al.*, 2014a). Two studies analyzed deduplication via UIDs (DOI, PMID, and arXiv ID) and other match key elements in Mendeley ( Hammerton *et al.*, 2012) and for a metasearch engine which covered five biomedical datadases ( Jiang *et al.*, 2014). One study created a citation index and used DOIs and other match key elements to match citing and cited publications ( Kim & Kim, 2013). One report tested the interoperability between Researchfish and local CRISs via DOI or PMID ( Research Councils UK, 2015). Finally, two studies reported on missing citations in WoS and Scopus. DOI was used to match identical publications in the two databases ( Franceschini *et al.*, 2013; Franceschini *et al.*, 2014). Nine studies, in addition to the coverage of UIDs, also addressed the precision or types of errors when UIDs are used as match keys. This information is central for our study. The types of errors are summarized in Table 1. As shown above the studies have used UIDs for different purposes. Still, we recognized the error types as relevant for our objective.

### Results of the case study

In the case study we analyzed research publications (co-)authored by the Department of Clinical Medicine (DoCM) at the University of Copenhagen, published in 2014, and registered and validated in Pure. We evaluated the matching process based on information about coverage, precision, and characteristics of publications matched versus not matched with UIDs as the match keys to detect errors, if any, in the matching process.

The share of publications matched between Pure and SciVal, or the coverage, is 85.6% in August and 89.3% in December.

There are precision issues for a minor part of the publication sets. Three of the error types reported by other studies ( Table 1) are also present in our publication sets.

Duplicate DOIs ( Table 1): An automatic report from SciVal states that 1837 publications (August) and 1876 publications (December) are matched with our Pure publication sets. These numbers are inflated due to DOI duplicates ( Table 2).

**Table 2.** Number and type of DOI duplicates in SciVal publication sets.

	August	December
Matched unique publications DOI duplicates in SciVal Matched publications including duplicates	1770 67 1837	1844 32 1876
Types of duplicates - articles-in-press/published articles - articles/articles - articles/articles with wrong DOI in Scopus and not in our Pure publication set - articles/same publications but recorded as another publication type Total	50 12 5 0 67	20 7 2 3 32

From August to December the number of duplicates decreases partly due to Scopus’s automatic cleaning process, where an Article in Press is deleted after the published version is registered in Scopus. We have discussed our results with consultants from Elsevier’s SciVal team and this has led to a correction of some of the other duplicates. It may also have had an effect that Elsevier in October 2015 started adding EIDs automatically to publication records in Pure.

Incorrect DOIs in reference lists and databases & DOIs not registered by the database where a bibliometric analysis is performed ( Table 1): In the August and December publication sets, respectively 5 and 2 of the DOI duplicates are examples of publications assigned a wrong DOI in Scopus ( Table 2). For a 10% sample of the remaining matched publications in the August and December publication sets we verified the DOIs. The publications were sorted by DOI and every tenth publication was searched in Scopus, PubMed, and CrossRef where title, authors, journal, and start page were compared. No errors were identified, but two publications did not have their DOIs registered in Scopus. Furthermore, we checked the 77 publications not matched in SciVal in August but matched in December. Of these, 36 publications have a DOI in our Pure publication set. No errors were found in Scopus. But as the publications were unmatched in August, DOIs or other UIDs must have been missing or been incorrect in Scopus in August or the publications were not indexed in Scopus in August.

We now turn to the characteristics of the publications in our publication sets. In Table 3– Table 7, the general characteristics of publications matched versus not matched in the integration between Pure and SciVal are presented. We have a particular interest in the publications’ UIDs as these are essential for a possible match. Publication type and language can give us an indication of whether all potential matches are made. We expected journal publications in English to be matched because they are well-covered in Scopus/SciVal. Table 3 gives an overview of how many publications were matched and unmatched. For the unmatched publications we also show how many have a UID.

**Table 3.** Export from Pure to SciVal - number of matched publications, unmatched publications with UID, and unmatched publications without UID. Download from August and December 2015.

	August 2068 publications		December 2066 publications	
Matched	1770	86%	1844	89%
Unmatched with UID	244	12%	178	9%
Unmatched without UID	54	3%	44	2%

In Table 4a & Table 4b we focus on the types of UIDs for the matched and the unmatched publications.

**Table 4a.** UID type for publications with UID exported from Pure to SciVal and matched. Download from August and December 2015.

	August 1770 publications		December 1844 publications	
DOI	1726	98%	1757	95%
PMID	1659	94%	1720	93%
EID	9	1%	-	-
Any UID	1770	100%	1844	100%

DOI is the most common UID ( Table 4a) but nearly as many publications have a PMID. This was expected as the majority of the publications were imported from PubMed to Pure in our specific publication set. In the August publication set, very few publications had an EID; most likely because Scopus is not commonly used for import to Pure by DoCM. In the December set we could not analyze the EIDs, as automatically attributed EIDs are not shown in our Pure reports of raw data. According to this report, 10 publications had an EID. But at least 32 additional publications in our Pure publication set from December had an EID as no other UID is assigned to them in our Pure raw data and they were matched in SciVal.

The unmatched publications with a UID are shown in Table 4b. PMID is the most common UID, up to 90%. Close to 40% of the publications have a DOI. For the December publication set, we assume that the unmatched publications have no EID, otherwise they should have been matched.

**Table 4b.** UID type for publications with UID exported from Pure to SciVal and not matched. Download from August and December 2015.

	August 244 publications		December 178 publications	
DOI	104	43%	71	40%
PMID	219	90%	155	87%
EID	1	0%	-	-
Any UID	244	100%	178	100%

In the following three tables we analyzed publication type as registered in Pure. Notable, but not surprising, is that close to 100% of matched publications are journal contributions ( Table 5a), as these are usually well-represented in Scopus/SciVal. What is surprising, however, is that practically the same percentage of unmatched publications with a UID is journal contributions ( Table 5b). For the publications without a UID ( Table 5c) there are still many journal publications, approximately 60%, but a much lower share than for the publications with a UID. The distributions among publication types do not differ substantially between the August and December publication sets. All publication sets include very few non-journal publications.

**Table 5a.** Publication type in Pure of publications with UID exported from Pure to SciVal and matched. Download from August and December 2015.

	August 1770 publications		December 1844 publications	
**Contribution to journal:**	**1765**	**>99%**	**1836**	**>99%**
Journal article	1592	90%	1650	89%
Letter	25	1%	25	1%
Review	102	6%	114	6%
Editorial	11	1%	12	1%
Comment/debate	35	2%	35	2%
Conference abstract in journal	0	0%	0	0%
**Book/anthology/thesis/report:**	**0**	**0%**	**2**	**<1%**
Book	0	0%	1	<1%
Anthology	0	0%	0	0%
Report	0	0%	0	0%
Doctoral thesis	0	0%	1	<1%
**Contribution to book/** **anthology/report:**	**4**	**<1%**	**4**	**<1%**
Book chapter	2	<1%	2	<1%
Article in proceedings	2	<1%	2	<1%
Encyclopedia chapter	0	0%	0	0%
**Contribution to conference:**	**0**	**0%**	**1**	**<1%**
Poster	0	0%	0	0%
Conference abstract for conference	0	0%	0	0%
Paper	0	0%	1	<1%
**Other**	**1**	**<1%**	**1**	**<1%**

**Table 5b.** Publication type in Pure of publications with UID exported from Pure to SciVal and not matched. Download from August and December 2015.

	August 244 publications		December 178 publications	
**Contribution to journal:**	**237**	**97%**	**172**	**97%**
Journal article	212	87%	157	88%
Letter	2	1%	2	1%
Review	20	8%	9	5%
Editorial	2	1%	2	1%
Comment/debate	0	0%	0	0%
Conference abstract in journal	1	<1%	2	1%
**Book/anthology/thesis/report:**	**1**	**<1%**	**0**	**0%**
Book	1	<1%	0	0%
Anthology	0	0%	0	0%
Report	0	0%	0	0%
Doctoral thesis	0	0%	0	0%
**Contribution to book/** **anthology/report:**	**6**	**2%**	**6**	**3%**
Book chapter	4	2%	4	2%
Article in proceedings	1	<1%	1	1%
Encyclopedia chapter	1	<1%	1	1%
**Contribution to conference:**	**0**	**0%**	**0**	**0%**
Poster	0	0%	0	0%
Conference abstract for conference	0	0%	0	0%
Paper	0	0%	0	0%
**Other**	0	0%	0	0%

**Table 5c.** Publication type in Pure of publications without UID not exported from Pure to SciVal. Download from August and December 2015.

	August 54 publications		December 44 publications	
**Contribution to journal:**	**33**	**61%**	**25**	**57%**
Journal article	22	41%	16	36%
Letter	2	4%	2	5%
Review	2	4%	1	2%
Editorial	1	2%	0	0%
Comment/debate	0	0%	0	0%
Conference abstract in journal	6	11%	6	14%
**Book/anthology/thesis/report:**	**7**	**13%**	**6**	**14%**
Book	0	0%	0	0%
Anthology	1	2%	1	2%
Report	1	2%	1	2%
Doctoral thesis	5	9%	4	9%
**Contribution to book/** **anthology/report:**	**8**	**15%**	**8**	**18%**
Book chapter	6	11%	6	14%
Article in proceedings	2	4%	2	5%
Encyclopedia chapter	0	0%	0	0%
**Contribution to conference:**	**5**	**9%**	**4**	**9%**
Poster	3	6%	3	7%
Conference abstract for conference	1	2%	1	2%
Paper	1	2%	0	0%
**Other**	1	2%	1	2%

We also analyzed the languages of the publications. Concerning the matched publications, 99% are written in English. Interestingly, the absolute number of matched publications in other languages increased from 4 to 27 between August and December ( Table 6a). Elsevier’s automatic assignment of EIDs may improve the match for these publications in our specific setting. However, our publication set is far too small to draw any conclusions from. For the unmatched publications with and without UID in the August and December publication sets, the ratios between English and other languages are close to fifty-fifty ( Table 6b and Table 6c).

**Table 6a.** Language of publications with UID exported from Pure to SciVal and matched. Download from August and December 2015.

	August 1770 publications		December 1844 publications	
English	1766	>99%	1817	99%
Other	4	<1%	27	1%

**Table 6b.** Language of publications with UID exported from Pure to SciVal and not matched. Download from August and December 2015.

	August 244 publications		December 178 publications	
English	127	52%	77	43%
Other	117	48%	101	57%

**Table 6c.** Language of publications without UID not exported from Pure to SciVal. Download from August and December 2015.

	August 54 publications		December 44 publications	
English	26	48%	23	52%
Other	28	52%	21	48%

**Table 7a.** Top journals according to number of publications, for publications with UIDs exported from Pure to SciVal and matched. Download from August and December 2015.

August 1770 publications		December 1844 publications	
**Journal title**	**Number of** **publications**	**Journal title**	**Number of** **publications**
*PLOS ONE*	62	*PLOS ONE*	65
*Contact Dermatitis*	27	*Danish Medical* *Journal*	38
*Danish Medical* *Journal*	27	*Contact Dermatitis*	27
*BMJ Open*	17	*BMJ Open*	19

**Table 7b.** Top journals according to number of publications for publications with UIDs exported from Pure to SciVal and not matched. Download from August and December 2015.

August 244 publications		December 178 publications	
**Journal title**	**Number of** **publications**	**Journal title**	**Number of** **publications**
*Ugeskrift for Læger,* *Ugeskrift for Laeger*	114	*Ugeskrift for Læger,* *Ugeskrift for Laeger*	99
*Danish Medical Journal*	10	*Clinical and Translational* *Allergy*	3
*Cochrane Database of* *Systematic Reviews*	7	*PLOS ONE*	3
*PLOS ONE*	6	*Annals of Clinical and* *Translational Neurology,* *EJNMMI Physics,* *Endocrine Connections,* *and Oncoimmunology* (all 2 publications each)	2

**Table 7c.** Top journals according to number of publications for publications without UIDs not exported from Pure to SciVal. Download from August and December 2015.

August 54 publications		December 44 publications	
**Journal title**	**Number of** **publications**	**Journal title**	**Number of** **publications**
*Ugeskrift for Læger,* *Ugeskrift for Laeger*	14	*Ugeskrift for Læger, Ugeskrift for* *Laeger*	8
*Clinical Nutrition*	3	*Clinical Nutrition*	3
*Early Intervention in* *Psychiatry*	3	*Early Intervention in Psychiatry*	3
*Journal of Anesthesia* *& Clinical Research*	2	*American Journal of Nuclear* *Medicine and Molecular Imaging,* *Annals of Internal Medicine,* *Annals of Sports Medicine and* *Research, Bibliotek for Laeger,* *European Respiratory Journal,* *International Journal of Anatomy* *and Research, Journal of* *Anesthesia & Clinical Research,* *Journal of Clinical Toxicology,* *Journal of Gastroenterology and* *Hepatology Research, Klinisk* *Sygepleje, Læring og Medier* *- LOM* (all 1 publication each)	1

Our analysis reveals more journal publications in English not matched in SciVal than we expected. Therefore we extracted lists of the top journals according to number of publications from our publication sets. For the unmatched publications a large share is published in the two journals of the Danish Medical Association ( *Ugeskrift for Læger* and *Danish Medical Journal*). Both are indexed by Scopus. Interestingly, we see *Ugeskrift for Læger* represented among the matched publications, the unmatched publications with UID, and the unmatched publications without UID. Also *PLOS ONE* publications are among both the matched and the unmatched publications, but not among publications without a UID. Three of six unmatched *PLOS ONE* publications from the August publication set are matched in December. The remaining three *PLOS ONE* publications were still not registered in Scopus in December. Table 7b and Table 7c includes more journals which are indexed by Scopus but the publications are not matched. For example *Clinical Nutrition* (cf. Table 7c) with 299 publications from 2014 indexed in Scopus, and *Clinical and Translational Allergy* (cf. Table 7b) with only 4 publications from 2014 indexed in Scopus. This may indicate some shortcomings in the Scopus indexing procedures. For our publication sets this seemed to be the biggest problem for a successful matching of publications with an UID. These results suggest that the missed matches were due to missing publications in Scopus. Another explanation could be missing UIDs in Scopus. This was detected in a study from 2012 (Bar-Ilan *et al.*, 2012, p. 101). In addition to this, 35 publications from the August and December publication sets were from journals not indexed by Scopus according to Scopus’ Content Coverage Guide.

In summary, the literature review shows that only a few studies report findings on UIDs as match keys. Results on coverage are reported and errors in the matching procedure are less frequently addressed ( Table 1).

The findings from the case study show that the majority of the publications were matched (85.6% in August and 89.3% in December). Almost all the matched publications have a DOI and are journal publications in English. Among the matched publications, 67 (3.8%) in the publication set from August have a duplicate DOI, whereas 32 (1.7%) from December do. Other error types ( Table 1) were observed which lowered the precision of the match between Pure and SciVal. Still, duplicate DOIs are the most prevalent problem. However, both coverage and precision have improved from August to December. This can be explained to some extent by Scopus’s automatic merging of Article in Press and the published version. Elsevier’s procedure of adding EIDs to publications in Pure may correct other duplicates and improve the coverage. Finally, duplicates may have been corrected manually by Elsevier in Scopus.

The unmatched publications also include journal publications. Close to half of these are in Danish and published in the journal *Ugeskrift for Læger* of which the indexing in Scopus is highly irregular. Our analysis indicates that journals with publications in English also suffer from similar irregular indexing but to a much lesser extent.

The matching of publications across databases can be used to create publication sets for bibliometric analysis. As an example we now briefly discuss how publication sets formed by using UIDs as match keys may affect the bibliometric indicators number of publications, number of citations, and the average number of citations per publication. This is to our knowledge only discussed briefly in the two studies. They both conclude that duplicate DOIs can lead to errors in bibliometric analysis ( Franceschini *et al.*, 2015, p. 2186; Valderrama-Zurián *et al.*, 2015, p. 575).

The coverage can affect bibliometric indicators. Results from our case study indicated that the majority of the publications from Pure are matched correctly in SciVal. Yet, the difference between the August and the December publication sets and the analysis of top journals ( Table 7a– Table 7c) show that coverage can be improved. This means that the number of publications and citations could be higher in a bibliometric analysis based on our publication set. We do not know the number of citations for the publications not matched but based on our knowledge about journals not indexed fully by Scopus we discuss the potential consequences for number of citations. *Ugeskrift for Læger* has over 100 publications that are not covered in Scopus/SciVal. The journal is not highly cited (Scopus 2014 IPP = 0.127, SNIP = 0.109) so inclusion of the missing publications would probably increase the number of citations a little, but lower the average number of citations per publication. However, inclusion of the missing publications for other journals could potentially have the opposite effect and increase the average number of citations per publication. An example is *PLOS ONE* (Scopus 2014 IPP = 3.270, SNIP = 1.034).

The precision of a bibliometric indicator is distorted by the fact that some DOIs are matched multiple times in SciVal. In most cases it is due to a duplicate of the same publication, but we also observed instances of publications in our Pure publication set with a DOI duplicate in SciVal not present in the Pure set ( Table 2). The duplicates have several implications for the bibliometric indicators number of publications, number of citations, and the average number of citations per publication:

The number of publications becomes inflated by inclusion of duplicates. In our publication sets from August and December the publication count increased by 3.8% and 1.7%, respectively. Therefore we recommend that when the number of publications is calculated, duplicates should be removed whether the duplicate publication is in the original Pure publication set or not.

Before citations are counted, all duplicates not present in the Pure publication set must be deleted. For the remaining duplicate pairs we found that sometimes both duplicates were cited independently. In all instances except one there was no overlap between the citations. Citations divided between duplicates in Scopus are also reported in another study where variations of a journal name results in duplicates in Scopus. It is suggested that databases like Scopus can improve verification of DOIs to solve the duplicate problem ( Valderrama-Zurián *et al.*, 2015). Publications were from 2014 and the export from Pure to SciVal was done in August and December 2015. So the publications had a very short time to attract citations. In total the publication set from August had 4,982 citations, but if citations for the duplicates were included the number was 5,047. This is an additional 1.3% citations. For the publication set from December the total number of citations has increased to 7,695, and to 7,720 if citations for duplicates are included. The duplicates account for 0.3% additional citations. In our study, many of the duplicates removed were Article in Press whereas the Article version was kept.

The calculation of the average number of citations per publication should not include duplicates in counting publications but include duplicates in counting citations. If duplicates are kept the average number of citations per publication will be too low. If the duplicates are removed some of the citations may also be discarded and again the average number of citations per publication will be too low.

Data of Evaluation of unique identifiers used as keys to match identical publications in Pure and SciVal Data sets consisting of publications from the Department of Clinical Medicine (DoCM) at University of Copenhagen (UCPH) are provided. A description of each file is provided in ‘Dataset description’. Click here for additional data file. Copyright: © 2016 Madsen HH et al. 2016 Data associated with the article are available under the terms of the Creative Commons Zero "No rights reserved" data waiver (CC0 1.0 Public domain dedication).

## Conclusion

UIDs are seen as effective keys to match identical publications across databases or identify duplicates in a database. The use of UIDs as match keys is well-implemented in many settings but only few studies evaluate how UIDs work as match keys. As DOIs are implemented in more and more settings DOI also becomes increasingly interesting as a match key. According to the publication years of the studies in our literature review we suggest that this trend took off around 2010.

Our case study confirms the findings of the literature review. UIDs as match keys do not return a 100% coverage of a publication set, and include errors for a small part of the matches. It is not possible to draw conclusions on when the coverage and precision is satisfactory as this should be discussed in relation to the purpose of a matching exercise, exemplified here as a bibliometric analysis.

In our case study we identified irregular indexing of journals in Scopus as the main problem for an optimal coverage. Duplicate DOIs were a particular problem for the precision of UIDs as match keys. This type of error is easy to detect while other types of errors demand a more thorough analysis of the publication sets. This analysis could be done by using a traditional match key based on title, author name, etc. Other error types also present in our case study are: incorrect UIDs in reference lists and databases, and UIDs not registered by the database where a bibliometric analysis is performed.

Match keys to detect identical publications across databases are used for many purposes, but our focus is bibliometric indicators. Here the duplicate DOIs constitute a problem as both keeping them in the publication set to improve the reliability of citation counts and deleting them to improve the reliability of publication counts will distort the calculation of average number of citations per publication and the many other bibliometric indicators which combine publication and citation counts. Also the coverage of a publication set can affect bibliometric indicators. We have discussed that failing to fully cover a low or high impact journal may also lead to imprecise bibliometric indicators.

## Future implications

Our purpose has been to contribute to the discussion on how well UIDs work as match keys for publications with a focus on preparing publication sets for bibliometric analysis. Compared to traditional match keys where bibliographic information is used, UIDs are efficient, but they also have drawbacks.

The coverage of UIDs is fully dependent on whether a UID is assigned to a publication, and its representations in publication lists and databases. Here the traditional match key has an advantage as it often is dependent on basic bibliographic data and can be modified to fit different formats. The traditional match key will probably have a good chance of retrieving all publications with a UID if the representations of the publications have basic bibliographic data of a fair quality. In addition, the traditional match key can retrieve publications without UIDs.

The precision of UIDs depends on how carefully a UID is assigned to a publication and its representations in publication lists and databases. Using a single UID as a match key can be fragile as no crosschecks are made on other data fields. Detection of errors requires an examination of the result of the matching process. The traditional match key often relies on more data fields and thus has a built-in crosscheck. Neither of the match keys will solve the problem of duplicates of identical publications.

We recommend more studies to be done on the pros and cons of UIDs because UIDs are being increasingly introduced for more entities and adopted as efficient match keys. The availability of UIDs may become critical for the inclusion of a publication or a database in a bibliometric analysis.

## Data availability

The data referenced by this article are under copyright with the following copyright statement: Copyright: © 2016 Madsen HH et al.

Data associated with the article are available under the terms of the Creative Commons Zero "No rights reserved" data waiver (CC0 1.0 Public domain dedication). 

F1000Research: Dataset 1. Data of evaluation of unique identifiers used for citation linking, 10.5256/f1000research.8913.d126923 ( Gauffriau *et al.*, 2016).
