# Supplementary material for: Evaluation of unique identifiers used as keys to match identical publications in Pure and SciVal – a case study from health science
Source: F1000Res. 2016 Sep 6;5:1539. Originally published 2016 Jun 29. [Version 2] doi: 10.12688/f1000research.8913.2 (PMC5017295; doi:10.12688/f1000research.8913.2)
Supplement: Peer review history

# Peer review: Referee response for version 2

**DOI:** 10.5256/f1000research.10259.r16140

**Published:** 2016-09-23

**Authors:** Keith G. Jeffery

**Affiliations:** 1 Keith G. Jeffery Consultants, Faringdon, UK

The authors have addressed the criticisms made adequately (and also those of the other referee) and so I approve of the article being indexed.

I have read this submission. I believe that I have an appropriate level of expertise to confirm that it is of an acceptable scientific standard.

# Peer review: Referee response for version 1

**DOI:** 10.5256/f1000research.9591.r15520

**Published:** 2016-09-02

**Authors:** Sarah L. Shreeves

**Affiliations:** 1 University of Miami, Coral Gables, FL, USA

This article describes a small study to better understand how unique identifiers (in this case DOIs) assigned to publications work as a match key for what the authors call 'citation linking' or matching identical publications in different databases in order to form a publication set that can then be used for bibliometric analysis. The study's goals were to lay out the characteristics of publications that were matched versus those that were unmatched, and to illustrate how the publications set formed might be affected by problems in the matching.

- I found the discussion of the citation linking throughout the article confusing, in part because my expectation of what that means is quite different than what is described in the article. My understanding of citation linking is that one is following works cited in a publication via the UID. It took several reads to clarify this; it may be something that the authors wish to clarify further in the abstract or introduction.
- I found the study design, methods, and conclusions to be adequate for the study particularly for the first objective (given the acknowledgement that this is not generalizable). It would be useful to expand this study to other disciplines to see whether there are similar issues and characteristics. In order to replicate this study in another discipline, however, more attention may need to be paid to coverage in Pure and SciVal, or use of other databases may need to be deployed.
- The second objective- to better understand how bibliometric analysis of the publication set formed might be skewed by issues in matching- was undermined by the size of the dataset- there seemed to be too little data from which to draw firm conclusions. However, given that, the description of the results are fine. 
- I believe that the Future Implications section provides the clearest implications of the study - essentially that UIDs aren't panaceas for deduplication/ citation matching/ etc.- and that use of UIDs as match points should be informed by potential points of failure. This description could have clarified other pieces of the article.

I wavered between 'Approved' and 'Approved with Reservations', and settled on 'Approved'. I believe that the revisions necessary are really clarity of language and noting throughout that the size and scope of the case study provide a limited window.

I have read this submission. I believe that I have an appropriate level of expertise to confirm that it is of an acceptable scientific standard.

# Response

**Published:** 2016-09-19

**Authors:** Marianne Gauffriau

**Affiliations:** University of Copenhagen

Thank you for your review. You touch upon important points. Please see our response below.

**Sarah L. Shreeves**: “I found the discussion of the citation linking throughout the article confusing, in part because my expectation of what that means is quite different than what is described in the article. My understanding of citation linking is that one is following works cited in a publication via the UID. It took several reads to clarify this; it may be something that the authors wish to clarify further in the abstract or introduction.”

**The term *citation linking***: In the literature we saw an inconsistent use of terms, but settled on "citation linking" to mean the matching of representations of identical publications across databases. We got similar comments from other reviewers and have edited our article, minimizing the term "citation linking", instead using "match key" and "matching process".

**Sarah L. Shreeves**: “I found the study design, methods, and conclusions to be adequate for the study particularly for the first objective (given the acknowledgement that this is not generalizable). It would be useful to expand this study to other disciplines to see whether there are similar issues and characteristics. In order to replicate this study in another discipline, however, more attention may need to be paid to coverage in Pure and SciVal, or use of other databases may need to be deployed.”

**Generalization**: We have focused on Pure and SciVal in this study and plan to do the same in a follow-up study where we expand the data sets to cover more research fields within health sciences and investigate a new matching process introduced by Elsevier. We did not consider including other databases as the main focus in the present study is the matching process. Thus we have selected a case where we expected the coverage to be good. If other disciplines with a low coverage in SciVal were studied, we agree that it would be important to analyze and discuss the coverage in more detail. As pointed out in your review, additional databases could be considered.

**Sarah L. Shreeves**: “The second objective- to better understand how bibliometric analysis of the publication set formed might be skewed by issues in matching- was undermined by the size of the dataset- there seemed to be too little data from which to draw firm conclusions. However, given that, the description of the results are fine.“

**Bibliometric indicators** and the discussion of them are based on a limited data set. We agree: Our results apply to our case alone and may serve as inspiration for other similar studies. In version 2 of the article the part on bibliometric indicators is elaborated upon. See the subsection *Results of the case study* in the paper, version 2.

**Sarah L. Shreeves**: “I believe that the Future Implications section provides the clearest implications of the study - essentially that UIDs aren't panaceas for deduplication/ citation matching/ etc.- and that use of UIDs as match points should be informed by potential points of failure. This description could have clarified other pieces of the article.”

**Future Implications**: In the section *Future Implications* we discuss UIDs as match key compared to a traditional match key. We hope to address this in a follow-up study, following Elsevier changing the way publications are matched between (Scopus and) Pure and SciVal: Publications in Pure are matched with publications in Scopus using traditional match keys and assigned a Scopus ID of the matched publication. The match between Pure and SciVal now happens using Scopus ID as only match key.    

**Sarah L. Shreeves**: “I wavered between 'Approved' and 'Approved with Reservations', and settled on 'Approved'. I believe that the revisions necessary are really clarity of language and noting throughout that the size and scope of the case study provide a limited window.”

 Again thank you for your review. We hope that our response and the edited paper have addressed your comments.

# Peer review: Referee response for version 1

**DOI:** 10.5256/f1000research.9591.r15523

**Published:** 2016-08-08

**Authors:** Keith G. Jeffery

**Affiliations:** 1 Keith G. Jeffery Consultants, Faringdon, UK

This paper aims to contribute to the discussion on use of UIDs for citation linking. It states two objectives:

1. Explore the coverage, precision, and characteristics of publications matched versus not matched with UIDs as the match key.
2. Illustrate how publication sets formed by using UIDs as the match key may affect the bibliometric indicators: Number of publications, number of citations and the average number of citations per publication.

The paper usefully lists the common UIDs used for research publications, and in so doing illustrates the problem of disjoint UID sets (intersected by other attributes of the publication) and lack of universal uniqueness but without commenting upon it. An important facility in PURE (based on CERIF) is the ability to utilise 'federated IDs' so that there can be several 'unique identifiers' for the same object thus allowing crosswalking within the metadata for a given publication. The paper mentions UIDs for publications and persons but not for organisations; the use of such UIDs is becoming more common and is useful for comparative analysis of performance by research organisational units.

 The paper states (P3) "Opposite to the other UIDs, the DOI is assigned to the publication itself and not merely to the publication’s representation in a database". There is a lack of clarity of thinking here. The DOI is a digital identifier and thus is not assigned to the publication itself (the scholarly content) but to a digital representation (textual, diagrammatic, tabular...) of it. The representation in the database is of two parts: the metadata (traditionally the library catalog entry) and the publication content (possibly with added datasets, software or artifacts). Throughout the paper it should be made clear that the work is based on the metadata - although the citation is within the textual content of the publication referring to a reference at the end of the publication from where a link can/should be made to the full text of that (cited) article. In fact ideally the link should have a time period of validity and semantics of the kind of citation (such as negative or positive, explanatory...).

 The paper states (P4) "The matching of identical publications in different databases is called citation linking or reference linking. Matching within the same database is called deduplication. The term citation matching is also used, but often for the more specific purposes where citing and cited publications are matched". It is important that the paper clarifies the thinking here also.  Citation has nothing to do with matching or de-duplication, although 'clean' data is required for effective and efficient citation. In fact the paper does not really discuss citation itself but only the utilisation of UIDs to achieve citation - and more particularly the difficulties of obtaining 'clean' data.

 The paper does not reference "Citation Linking: Improving Access to Online Journals"

 S. Hitchcock*, L. Carr, S. Harris, J. M. N. Hey and W. Hall *Proceedings of the 2nd ACM International Conference on Digital Libraries*, edited by Robert B. Allen and Edie Rasmussen, 1997 (New York, USA: Association for Computing Machinery), pp. 115-122 available at http://journals.ecs.soton.ac.uk/acmdl97.htm  which states clearly (and as early as 1997):

 "The likely effect of citation linking can be gauged by recognising a direct parallel with citation indexing, which is sometimes referred to as 'forward' referencing (i.e. for a given article, all the subsequent papers that cite it), developed by Garfield (1955). Garfield's description of citation indexing as an 'association of ideas' bears remarkable similarity to Bush's 'association of thoughts' which anticipated modern hypertext. Citation linking combines the two approaches, mapping both reference data and citation index data on to the text in the form of links. Adding electronic links to the literature is introducing a new culture in many respects, but citation linking is likely to be acceptable to the academic community because it builds on practices established in other forms such as print. It also allows the community to exploit its own intellectual input in this process, recognised in Garfield's original rationale that "by using authors' references in compiling the citation index, we are in reality utilizing an army of indexers"."   Here citation linking is used - and with precedence - to mean something quite different to the use in this paper.  

 The literature review is appropriate. The case study is limited in organisational unit, research discipline, time period; however the results are useful and could - with advantage - be compared with similar studies in other disciplines /organisational units and time periods.

 Overall the paper is a useful contribution and hopefully will stimulate similar studies in other disciplines and time ranges.  but the lack of clarity of thinking should be addressed. I would suggest:

1. a paragraph clarifying the inter-relationships of metadata and content for one publication with that for another cited from the first;
2. a paragraph explaining citation, citation linking, citation indexing;
3. the existing paragraph on UIDs being expanded to explain the usefulness of 'federated IDs' for crosswalking within the metadata of a given publication.

I have read this submission. I believe that I have an appropriate level of expertise to confirm that it is of an acceptable scientific standard, however I have significant reservations, as outlined above.

# Response

**Published:** 2016-08-25

**Authors:** Marianne Gauffriau

**Affiliations:** University of Copenhagen

**Response to Marion Schmidt and Keith G. Jeffery**

 Thank you for the valuable reviews. Below we quote the issues raised in the reviews followed by our response and description of how we have edited the paper. Please note that we have sent the same reply to both of you so you have the same information on how we have edited the paper.

**Marion Schmidt**: “In the introduction and literature review, it should be made clearer that the authors use a specific definition of the term citation linking (linking items between databases) for their study and it should be clarified if other studies refer to the same or other scenarios (like reference matching or deduplication).”

**Keith G. Jeffery**: “The paper states (P4) "The matching of identical publications in different databases is called citation linking or reference linking. Matching within the same database is called deduplication. The term citation matching is also used, but often for the more specific purposes where citing and cited publications are matched". It is important that the paper clarifies the thinking here also.  Citation has nothing to do with matching or de-duplication, although 'clean' data is required for effective and efficient citation. In fact the paper does not really discuss citation itself but only the utilisation of UIDs to achieve citation - and more particularly the difficulties of obtaining 'clean' data.

 The paper does not reference "Citation Linking: Improving Access to Online Journals"

 S. Hitchcock*, L. Carr, S. Harris, J. M. N. Hey and W. Hall Proceedings of the 2nd ACM International Conference on Digital Libraries, edited by Robert B. Allen and Edie Rasmussen, 1997 (New York, USA: Association for Computing Machinery), pp. 115-122 available at http://journals.ecs.soton.ac.uk/acmdl97.htm  which states clearly (and as early as 1997):

 "The likely effect of citation linking can be gauged by recognising a direct parallel with citation indexing, which is sometimes referred to as 'forward' referencing (i.e. for a given article, all the subsequent papers that cite it), developed by Garfield (1955). Garfield's description of citation indexing as an 'association of ideas' bears remarkable similarity to Bush's 'association of thoughts' which anticipated modern hypertext. Citation linking combines the two approaches, mapping both reference data and citation index data on to the text in the form of links. Adding electronic links to the literature is introducing a new culture in many respects, but citation linking is likely to be acceptable to the academic community because it builds on practices established in other forms such as print. It also allows the community to exploit its own intellectual input in this process, recognised in Garfield's original rationale that "by using authors' references in compiling the citation index, we are in reality utilizing an army of indexers"."   Here citation linking is used - and with precedence - to mean something quite different to the use in this paper.

 […]

 I would suggest:

 […]

 2. a paragraph explaining citation, citation linking, citation indexing;”

**The term *citation linking*** has caused confusion as citation can be understood as the reference from one paper (citing document) to another paper (cited document). Also in the literature we see an inconsistent use of the term. By citation linking we have meant the linking of representations of identical publications across databases. We have had difficulties finding an unambiguous term for this and ended with citation linking. We now have minimized the use of the term citation linking. Instead we use match key and matching process.

**Marion Schmidt**: “The sample is a convenience sample based only on one year and one subject field (health sciences), with a predominantly journal- and English-based publication culture. The authors describe their first objective as "Explore the coverage, precision, and characteristics of publications matched versus not matched with UIDs as the match key", after introducing the more general goal to investigate "how well UIDs work for citation linking" (p.3). In order to truly answer the goals and objectives, the hypothesis should be discussed and tested that implementation of UIDs may vary across disciplines, depending on the amount of e.g. smaller, regional journals and easy data import options for a system like Pure from, e.g. PubMed. In this perspective a careful sampling strategy representative for all subjects that are, to a varying extent, covered in the target database SciVal/Scopus as well as more publication years, would have been much more fruitful in order to assess the coverage and thus usability of UIDs in both systems. Thus, data from a university-wide implementation of Pure would make up a reasonable case study.

 The authors deal inconsistently with this issue as they try to rectify their sample on the one hand "As this type of publication and the health sciences are well-covered in Scopus/SciVal [...], we expected the DoCM publication set to be well-fitted for our purpose, namely to explore the citation linking process, rather than how well SciVal covers publications from a department" (p.4) - which is a non-adequate argument as the coverage could be considered separately. On the other hand, they concede that their "case study" may not lead to generalizable results and that results will therefore be compared to those from a literature review (p.4).

 […]

 I would strongly suggest expanding the initial sample to other fields.”

**Keith G. Jeffery**: “The case study is limited in organisational unit, research discipline, time period; however the results are useful and could - with advantage - be compared with similar studies in other disciplines /organisational units and time periods.”

**The case** we work with was deliberately chosen as the best scenario and not as a sample fit for generalization. We wanted to study how well UIDs work as match key in the integration between Pure and SciVal, so it seemed beneficial to work with a sample with as many UIDs registered as possible. Because of the attributes of publications from the Department of Clinical Medicine (journal publications in English), they seemed likely to have a high number of UIDs registered. Furthermore, the integration between Pure and SciVal is relatively new and we only had limited knowledge about how it worked and found no evaluations of the integration in the literature. We know that publication sets from health sciences are well-covered by Scopus. With the potential good coverage between Pure and SciVal we hoped that our publication set would allow us primarily to focus on the matching itself. The coverage of SciVal/Scopus is reported in the paper, but this is supporting information.

 In the selection of the case we also took local conditions into account. Working with Pure data validated by the University Library, that is publications from 2014, we felt confident that the sample would have fewer errors regarding registration/correct registration of UIDs than samples from previous years where no central validation routines were in place.

 It is not possible for us to repeat our analysis with a case covering more research areas and more publication years, as the matching process for the integration between Pure and SciVal has changed. Now only Scopus ID is used as match key. Before, DOI and PubMed ID also worked as match keys. However, we are doing a follow-up study to see how the new matching process works for our original publication set and a new larger publication set.

**Marion Schmidt**: “Thus, title and abstract information do - in my opinion - not reflect the actual limits of the study adequately.”

**The objective** was formulated somewhat broadly and could give the impression that the study included a thorough analysis of the coverage of SciVal/Scopus and the study yielded results which could be generalized to other

 research fields. As explained above, this is not the case. We have specified the objective to clearly show that we evaluate how well UIDs work as match key. Our focus is the matching process and any errors in this process. Furthermore, we do only work with one case – the integration between Pure and SciVal for a publication set from health sciences. The case cannot be generalized to other research fields or settings. As a consequence of the reformulated objective, we have changed the title and abstract accordingly.

**Keith G. Jeffery**: “The paper usefully lists the common UIDs used for research publications, and in so doing illustrates the problem of disjoint UID sets (intersected by other attributes of the publication) and lack of universal uniqueness but without commenting upon it. An important facility in PURE (based on CERIF) is the ability to utilise 'federated IDs' so that there can be several 'unique identifiers' for the same object thus allowing crosswalking within the metadata for a given publication. The paper mentions UIDs for publications and persons but not for organisations; the use of such UIDs is becoming more common and is useful for comparative analysis of performance by research organisational units.

 The paper states (P3) "Opposite to the other UIDs, the DOI is assigned to the publication itself and not merely to the publication’s representation in a database". There is a lack of clarity of thinking here. The DOI is a digital identifier and thus is not assigned to the publication itself (the scholarly content) but to a digital representation (textual, diagrammatic, tabular...) of it. The representation in the database is of two parts: the metadata (traditionally the library catalog entry) and the publication content (possibly with added datasets, software or artifacts). Throughout the paper it should be made clear that the work is based on the metadata - although the citation is within the textual content of the publication referring to a reference at the end of the publication from where a link can/should be made to the full text of that (cited) article. In fact ideally the link should have a time period of validity and semantics of the kind of citation (such as negative or positive, explanatory...).

 […]

 I would suggest:

 1. a paragraph clarifying the inter-relationships of metadata and content for one publication with that for another cited from the first;

 […]

 3. the existing paragraph on UIDs being expanded to explain the usefulness of 'federated IDs' for crosswalking within the metadata of a given publication.”

**The description of a publication and its UIDs **is elaborated. We did not analyze the full content of a publication but only parts of its metadata representations in Pure and SciVal. A publication can have more UIDs assigned to it in one or both of the databases. We assumed that each of the UIDs for a publication in a database represented the same publication. See *Introduction* and the subsection *Limitations* in the paper, version 2.

**Marion Schmidt**: “On the other hand, they concede that their "case study" may not lead to generalizable results and that results will therefore be compared to those from a literature review (p.4). This claim, however, is not really fulfilled, as the studies mentioned in the literature review use UIDs for different purposes, constellations and databases. More importantly, no real comparison takes place, as the authors only recap "but all conclude that UIDs do not cover all records in the databases" (p.6). In order to contextualize their own results, concrete settings and results of other studies should be represented and discussed.

 […]

 In the introduction and literature review, it should be made clearer that the authors use a specific definition of the term citation linking (linking items between databases) for their study and it should be clarified if other studies refer

 to the same or other scenarios (like reference matching or deduplication).”

**Keith G. Jeffery**: “The literature review is appropriate.”

**The literature review** shows the big picture and now also includes more details on the studies included. None of the studies in the review were directly comparable to our study as we analyzed how well UIDs work as match key in the matching process between Pure and SciVal. We now show that the studies in the review used UIDs for many different matching processes. Only two of the studies focused on the evaluation of UIDs. Still, based on all the studies we summarized information on error types when UIDs are used as match keys, which is relevant for our study. See the subsection *Results of the literature review* in the paper, version 2.

**Marion Schmidt**: “With regard to the second purpose "Illustrate how publication sets formed by using UIDs as the match key may affect the bibliometric indicators: Number of publications, number of citations and the average number of citations per publication", the authors do not actually calculate citation indicators including and excluding publications (which are covered, but could not be matched via UIDs), but discuss the problem mostly theoretically.”

**The number of citations **was only calculated for the publications matched in SciVal at the time we did the exports from Pure to SciVal (August and December 2015). We did not have the number of citations for the other publications. This information is added to the article. For the matched publications we have added the number of citations including and excluding duplicates. See the subsection *Results of the case study* in the paper, version 2 *.*

**Marion Schmidt**: “Methods and data (with exceptions mentioned separately below) are sufficiently clearly documented, but the whole study lacks generalizability and, partly, elaborateness of analyses, as in case of the citation indicator perspective. Besides, given the rather manageable amounts of unmatched publications in this study, a comprehensive and more elaborate search and analysis of the causes of the missed match (not covered, missing UID in Scopus, ..) would be preferable. In larger corpora, this could be done via a random sample.”

**Causes for missed matches for publications with a UID** were not investigated when we did the exports from Pure to SciVal at publication level but only at journal level (Table 7a-7c). We have looked into the question now, but many of the publications not matched in August and December 2015 are now matched. We added to the paper that from our analysis of journals (Table 7a-7c) it is likely that the publications not matched were not indexed in Scopus. We identified three *PLOS ONE* publications which were not indexed in Scopus, and *Clinical and Translational Allergy *had only four publications from 2014 indexed in Scopus. But the missed matches can also be due to missing UIDs in Scopus. For 35 publications we established that they were published in journals not indexed in Scopus. This is also added to the paper. See the subsection *Results of the case study* in the paper, version 2 *.*

**Marion Schmidt**: “The authors write "In the integration between Pure and SciVal DOI, PMID and EID are used as match keys for citation linking. From March 2016 the integration between Pure and SciVal is based on EID alone. This will not affect the present study as we analyze publication sets downloaded in August and December 2015" (p.3).

 The authors should discuss what this does mean with respect of the relevance of their results. Could it mean that the matching of current publications will be probably better?”

**The new matching process between Pure and SciVal** means that our study is a documentation of the first matching processes between Pure and SciVal and can serve as background for new evaluations. We have added this point to the paper. We do not know if the new functionality is better than the ones we have evaluated but we are looking into that in a follow-up study. See the *Introduction* in the paper, version 2  

**Marion Schmidt**: “Regarding the publication type categorization: Has the categorization been informed by some available classification or, e.g. database scheme? In my opinion, some mappings are sub-optimal and particular; especially the assignment of Doctoral Thesis to "Other" instead of "Book", Encyclopedia chapter to "Other" instead of "Contribution to Book", Editorial to "Other" instead of "Journal", Article in Proceedings to "Journal" instead of "Contribution to Book" (please compare the WoS document type and publication type classification for the third and fourth case). Including so many and different publication types in a residual category is unprofitable for the later analysis.”

**The categorization of publication types** has been reorganized according to the categories in Pure. See Table 5a-c.

**Marion Schmidt**: “I did not understand the fact that EIDs seem to have been attributed automatically without being visible in the raw data. How?”

**The EID automatically attributed to publications in Pure** was introduced with Pure version 4.23.0. EIDs are added to publications in Pure as an automatic job. In the beginning the job ran every day with a sub-set of the publications in order not to overload the matching service. 90 days after installation of the new version of Pure the job had covered all publications. Now when a new publications are added to Pure they will be included in the first coming match, hence within 14 days.

 The automatic attributed EIDs are not shown in our raw data (excel files downloaded from Pure). Therefore it is not possible to detect which publications are matched on the automatic attributed EIDs. More publications are matched in the November dataset, and we assume they are matched on the automatic attributed EID but cannot prove it.

 Once again thank you for the reviews. We hope that our response and the edited paper have addressed all your comments.

# Peer review: Referee response for version 1

**DOI:** 10.5256/f1000research.9591.r14675

**Published:** 2016-07-28

**Authors:** Marion Schmidt

**Affiliations:** 1 German Centre for Higher Education Research and Science Studies (DZHW), Berlin, Germany

The sample is a convenience sample based only on one year and one subject field (health sciences), with a predominantly journal- and English-based publication culture. The authors describe their first objective as "Explore the coverage, precision, and characteristics of publications matched versus not matched with UIDs as the match key", after introducing the more general goal to investigate "how well UIDs work for citation linking" (p.3). In order to truly answer the goals and objectives, the hypothesis should be discussed and tested that implementation of UIDs may vary across disciplines, depending on the amount of e.g. smaller, regional journals and easy data import options for a system like Pure from, e.g. PubMed. In this perspective a careful sampling strategy representative for all subjects that are, to a varying extent, covered in the target database SciVal/Scopus as well as more publication years, would have been much more fruitful in order to assess the coverage and thus usability of UIDs in both systems. Thus, data from a university-wide implementation of Pure would make up a reasonable case study.

 The authors deal inconsistently with this issue as they try to rectify their sample on the one hand "As this type of publication and the health sciences are well-covered in Scopus/SciVal [...], we expected the DoCM publication set to be well-fitted for our purpose, namely to explore the citation linking process, rather than how well SciVal covers publications from a department" (p.4) - which is a non-adequate argument as the coverage could be considered separately. On the other hand, they concede that their "case study" may not lead to generalizable results and that results will therefore be compared to those from a literature review (p.4). This claim, however, is not really fulfilled, as the studies mentioned in the literature review use UIDs for different purposes, constellations and databases. More importantly, no real comparison takes place, as the authors only recap "but all conclude that UIDs do not cover all records in the databases" (p.6). In order to contextualize their own results, concrete settings and results of other studies should be represented and discussed.

 With regard to the second purpose "Illustrate how publication sets formed by using UIDs as the match key may affect the bibliometric indicators: Number of publications, number of citations and the average number of citations per publication", the authors do not actually calculate citation indicators including and excluding publications (which are covered, but could not be matched via UIDs), but discuss the problem mostly theoretically.

 I would strongly suggest expanding the initial sample to other fields.

 Thus, title and abstract information do - in my opinion - not reflect the actual limits of the study adequately.

 Methods and data (with exceptions mentioned separately below) are sufficiently clearly documented, but the whole study lacks generalizability and, partly, elaborateness of analyses, as in case of the citation indicator perspective. Besides, given the rather manageable amounts of unmatched publications in this study, a comprehensive and more elaborate search and analysis of the causes of the missed match (not covered, missing UID in Scopus, ..) would be preferable. In larger corpora, this could be done via a random sample.

 In the introduction and literature review, it should be made clearer that the authors use a specific definition of the term citation linking (linking items between databases) for their study and it should be clarified if other studies refer to the same or other scenarios (like reference matching or deduplication).

 The authors write "In the integration between Pure and SciVal DOI, PMID and EID are used as match keys for citation linking. From March 2016 the integration between Pure and SciVal is based on EID alone. This will not affect the present study as we analyze publication sets downloaded in August and December 2015" (p.3).

 The authors should discuss what this does mean with respect of the relevance of their results. Could it mean that the matching of current publications will be probably better?

 Regarding the publication type categorization: Has the categorization been informed by some available classification or, e.g. database scheme? In my opinion, some mappings are sub-optimal and particular; especially the assignment of Doctoral Thesis to "Other" instead of "Book", Encyclopedia chapter to "Other" instead of "Contribution to Book", Editorial to "Other" instead of "Journal", Article in Proceedings to "Journal" instead of "Contribution to Book" (please compare the WoS document type and publication type classification for the third and fourth case). Including so many and different publication types in a residual category is unprofitable for the later analysis.

 I did not understand the fact that EIDs seem to have been attributed automatically without being visible in the raw data. How?

I have read this submission. I believe that I have an appropriate level of expertise to confirm that it is of an acceptable scientific standard, however I have significant reservations, as outlined above.

# Response

**Published:** 2016-08-25

**Authors:** Marianne Gauffriau

**Affiliations:** University of Copenhagen

**Response to Marion Schmidt and Keith G. Jeffery**

 Thank you for the valuable reviews. Below we quote the issues raised in the reviews followed by our response and description of how we have edited the paper. Please note that we have sent the same reply to both of you so you have the same information on how we have edited the paper.

**Marion Schmidt**: “In the introduction and literature review, it should be made clearer that the authors use a specific definition of the term citation linking (linking items between databases) for their study and it should be clarified if other studies refer to the same or other scenarios (like reference matching or deduplication).”

**Keith G. Jeffery**: “The paper states (P4) "The matching of identical publications in different databases is called citation linking or reference linking. Matching within the same database is called deduplication. The term citation matching is also used, but often for the more specific purposes where citing and cited publications are matched". It is important that the paper clarifies the thinking here also.  Citation has nothing to do with matching or de-duplication, although 'clean' data is required for effective and efficient citation. In fact the paper does not really discuss citation itself but only the utilisation of UIDs to achieve citation - and more particularly the difficulties of obtaining 'clean' data.

 The paper does not reference "Citation Linking: Improving Access to Online Journals"

 S. Hitchcock*, L. Carr, S. Harris, J. M. N. Hey and W. Hall Proceedings of the 2nd ACM International Conference on Digital Libraries, edited by Robert B. Allen and Edie Rasmussen, 1997 (New York, USA: Association for Computing Machinery), pp. 115-122 available at http://journals.ecs.soton.ac.uk/acmdl97.htm  which states clearly (and as early as 1997):

 "The likely effect of citation linking can be gauged by recognising a direct parallel with citation indexing, which is sometimes referred to as 'forward' referencing (i.e. for a given article, all the subsequent papers that cite it), developed by Garfield (1955). Garfield's description of citation indexing as an 'association of ideas' bears remarkable similarity to Bush's 'association of thoughts' which anticipated modern hypertext. Citation linking combines the two approaches, mapping both reference data and citation index data on to the text in the form of links. Adding electronic links to the literature is introducing a new culture in many respects, but citation linking is likely to be acceptable to the academic community because it builds on practices established in other forms such as print. It also allows the community to exploit its own intellectual input in this process, recognised in Garfield's original rationale that "by using authors' references in compiling the citation index, we are in reality utilizing an army of indexers"."   Here citation linking is used - and with precedence - to mean something quite different to the use in this paper.

 […]

 I would suggest:

 […]

 2. a paragraph explaining citation, citation linking, citation indexing;”

**The term *citation linking*** has caused confusion as citation can be understood as the reference from one paper (citing document) to another paper (cited document). Also in the literature we see an inconsistent use of the term. By citation linking we have meant the linking of representations of identical publications across databases. We have had difficulties finding an unambiguous term for this and ended with citation linking. We now have minimized the use of the term citation linking. Instead we use match key and matching process.

**Marion Schmidt**: “The sample is a convenience sample based only on one year and one subject field (health sciences), with a predominantly journal- and English-based publication culture. The authors describe their first objective as "Explore the coverage, precision, and characteristics of publications matched versus not matched with UIDs as the match key", after introducing the more general goal to investigate "how well UIDs work for citation linking" (p.3). In order to truly answer the goals and objectives, the hypothesis should be discussed and tested that implementation of UIDs may vary across disciplines, depending on the amount of e.g. smaller, regional journals and easy data import options for a system like Pure from, e.g. PubMed. In this perspective a careful sampling strategy representative for all subjects that are, to a varying extent, covered in the target database SciVal/Scopus as well as more publication years, would have been much more fruitful in order to assess the coverage and thus usability of UIDs in both systems. Thus, data from a university-wide implementation of Pure would make up a reasonable case study.

 The authors deal inconsistently with this issue as they try to rectify their sample on the one hand "As this type of publication and the health sciences are well-covered in Scopus/SciVal [...], we expected the DoCM publication set to be well-fitted for our purpose, namely to explore the citation linking process, rather than how well SciVal covers publications from a department" (p.4) - which is a non-adequate argument as the coverage could be considered separately. On the other hand, they concede that their "case study" may not lead to generalizable results and that results will therefore be compared to those from a literature review (p.4).

 […]

 I would strongly suggest expanding the initial sample to other fields.”

**Keith G. Jeffery**: “The case study is limited in organisational unit, research discipline, time period; however the results are useful and could - with advantage - be compared with similar studies in other disciplines /organisational units and time periods.”

**The case** we work with was deliberately chosen as the best scenario and not as a sample fit for generalization. We wanted to study how well UIDs work as match key in the integration between Pure and SciVal, so it seemed beneficial to work with a sample with as many UIDs registered as possible. Because of the attributes of publications from the Department of Clinical Medicine (journal publications in English), they seemed likely to have a high number of UIDs registered. Furthermore, the integration between Pure and SciVal is relatively new and we only had limited knowledge about how it worked and found no evaluations of the integration in the literature. We know that publication sets from health sciences are well-covered by Scopus. With the potential good coverage between Pure and SciVal we hoped that our publication set would allow us primarily to focus on the matching itself. The coverage of SciVal/Scopus is reported in the paper, but this is supporting information.

 In the selection of the case we also took local conditions into account. Working with Pure data validated by the University Library, that is publications from 2014, we felt confident that the sample would have fewer errors regarding registration/correct registration of UIDs than samples from previous years where no central validation routines were in place.

 It is not possible for us to repeat our analysis with a case covering more research areas and more publication years, as the matching process for the integration between Pure and SciVal has changed. Now only Scopus ID is used as match key. Before, DOI and PubMed ID also worked as match keys. However, we are doing a follow-up study to see how the new matching process works for our original publication set and a new larger publication set.

**Marion Schmidt**: “Thus, title and abstract information do - in my opinion - not reflect the actual limits of the study adequately.”

**The objective** was formulated somewhat broadly and could give the impression that the study included a thorough analysis of the coverage of SciVal/Scopus and the study yielded results which could be generalized to other research fields. As explained above, this is not the case. We have specified the objective to clearly show that we evaluate how well UIDs work as match key. Our focus is the matching process and any errors in this process. Furthermore, we do only work with one case – the integration between Pure and SciVal for a publication set from health sciences. The case cannot be generalized to other research fields or settings. As a consequence of the reformulated objective, we have changed the title and abstract accordingly.

**Keith G. Jeffery**: “The paper usefully lists the common UIDs used for research publications, and in so doing illustrates the problem of disjoint UID sets (intersected by other attributes of the publication) and lack of universal uniqueness but without commenting upon it. An important facility in PURE (based on CERIF) is the ability to utilise 'federated IDs' so that there can be several 'unique identifiers' for the same object thus allowing crosswalking within the metadata for a given publication. The paper mentions UIDs for publications and persons but not for organisations; the use of such UIDs is becoming more common and is useful for comparative analysis of performance by research organisational units.

 The paper states (P3) "Opposite to the other UIDs, the DOI is assigned to the publication itself and not merely to the publication’s representation in a database". There is a lack of clarity of thinking here. The DOI is a digital identifier and thus is not assigned to the publication itself (the scholarly content) but to a digital representation (textual, diagrammatic, tabular...) of it. The representation in the database is of two parts: the metadata (traditionally the library catalog entry) and the publication content (possibly with added datasets, software or artifacts). Throughout the paper it should be made clear that the work is based on the metadata - although the citation is within the textual content of the publication referring to a reference at the end of the publication from where a link can/should be made to the full text of that (cited) article. In fact ideally the link should have a time period of validity and semantics of the kind of citation (such as negative or positive, explanatory...).

 […]

 I would suggest:

 1. a paragraph clarifying the inter-relationships of metadata and content for one publication with that for another cited from the first;

 […]

 3. the existing paragraph on UIDs being expanded to explain the usefulness of 'federated IDs' for crosswalking within the metadata of a given publication.”

**The description of a publication and its UIDs **is elaborated. We did not analyze the full content of a publication but only parts of its metadata representations in Pure and SciVal. A publication can have more UIDs assigned to it in one or both of the databases. We assumed that each of the UIDs for a publication in a database represented the same publication. See *Introduction* and the subsection *Limitations* in the paper, version 2.

**Marion Schmidt**: “On the other hand, they concede that their "case study" may not lead to generalizable results and that results will therefore be compared to those from a literature review (p.4). This claim, however, is not really fulfilled, as the studies mentioned in the literature review use UIDs for different purposes, constellations and databases. More importantly, no real comparison takes place, as the authors only recap "but all conclude that UIDs do not cover all records in the databases" (p.6). In order to contextualize their own results, concrete settings and results of other studies should be represented and discussed.

 […]

 In the introduction and literature review, it should be made clearer that the authors use a specific definition of the term citation linking (linking items between databases) for their study and it should be clarified if other studies refer to the same or other scenarios (like reference matching or deduplication).”

**Keith G. Jeffery**: “The literature review is appropriate.”

**The literature review** shows the big picture and now also includes more details on the studies included. None of the studies in the review were directly comparable to our study as we analyzed how well UIDs work as match key in the matching process between Pure and SciVal. We now show that the studies in the review used UIDs for many different matching processes. Only two of the studies focused on the evaluation of UIDs. Still, based on all the studies we summarized information on error types when UIDs are used as match keys, which is relevant for our study. See the subsection *Results of the literature review* in the paper, version 2.

**Marion Schmidt**: “With regard to the second purpose "Illustrate how publication sets formed by using UIDs as the match key may affect the bibliometric indicators: Number of publications, number of citations and the average number of citations per publication", the authors do not actually calculate citation indicators including and excluding publications (which are covered, but could not be matched via UIDs), but discuss the problem mostly theoretically.”

**The number of citations **was only calculated for the publications matched in SciVal at the time we did the exports from Pure to SciVal (August and December 2015). We did not have the number of citations for the other publications. This information is added to the article. For the matched publications we have added the number of citations including and excluding duplicates. See the subsection *Results of the case study* in the paper, version 2 *.*

**Marion Schmidt**: “Methods and data (with exceptions mentioned separately below) are sufficiently clearly documented, but the whole study lacks generalizability and, partly, elaborateness of analyses, as in case of the citation indicator perspective. Besides, given the rather manageable amounts of unmatched publications in this study, a comprehensive and more elaborate search and analysis of the causes of the missed match (not covered, missing UID in Scopus, ..) would be preferable. In larger corpora, this could be done via a random sample.”

**Causes for missed matches for publications with a UID** were not investigated when we did the exports from Pure to SciVal at publication level but only at journal level (Table 7a-7c). We have looked into the question now, but many of the publications not matched in August and December 2015 are now matched. We added to the paper that from our analysis of journals (Table 7a-7c) it is likely that the publications not matched were not indexed in Scopus. We identified three *PLOS ONE* publications which were not indexed in Scopus, and *Clinical and Translational Allergy *had only four publications from 2014 indexed in Scopus. But the missed matches can also be due to missing UIDs in Scopus. For 35 publications we established that they were published in journals not indexed in Scopus. This is also added to the paper. See the subsection *Results of the case study* in the paper, version 2 *.*

**Marion Schmidt**: “The authors write "In the integration between Pure and SciVal DOI, PMID and EID are used as match keys for citation linking. From March 2016 the integration between Pure and SciVal is based on EID alone. This will not affect the present study as we analyze publication sets downloaded in August and December 2015" (p.3).

 The authors should discuss what this does mean with respect of the relevance of their results. Could it mean that the matching of current publications will be probably better?”

**The new matching process between Pure and SciVal** means that our study is a documentation of the first matching processes between Pure and SciVal and can serve as background for new evaluations. We have added this point to the paper. We do not know if the new functionality is better than the ones we have evaluated but we are looking into that in a follow-up study. See the *Introduction* in the paper, version 2  

**Marion Schmidt**: “Regarding the publication type categorization: Has the categorization been informed by some available classification or, e.g. database scheme? In my opinion, some mappings are sub-optimal and particular; especially the assignment of Doctoral Thesis to "Other" instead of "Book", Encyclopedia chapter to "Other" instead of "Contribution to Book", Editorial to "Other" instead of "Journal", Article in Proceedings to "Journal" instead of "Contribution to Book" (please compare the WoS document type and publication type classification for the third and fourth case). Including so many and different publication types in a residual category is unprofitable for the later analysis.”

**The categorization of publication types** has been reorganized according to the categories in Pure. See Table 5a-c.

**Marion Schmidt**: “I did not understand the fact that EIDs seem to have been attributed automatically without being visible in the raw data. How?”

**The EID automatically attributed to publications in Pure** was introduced with Pure version 4.23.0. EIDs are added to publications in Pure as an automatic job. In the beginning the job ran every day with a sub-set of the publications in order not to overload the matching service. 90 days after installation of the new version of Pure the job had covered all publications. Now when a new publications are added to Pure they will be included in the first coming match, hence within 14 days.

 The automatic attributed EIDs are not shown in our raw data (excel files downloaded from Pure). Therefore it is not possible to detect which publications are matched on the automatic attributed EIDs. More publications are matched in the November dataset, and we assume they are matched on the automatic attributed EID but cannot prove it.

 Once again thank you for the reviews. We hope that our response and the edited paper have addressed all your comments.
